# Apicoplast ribosomal protein S10-V127M enhances artemisinin resistance of a Kelch13 transgenic *Plasmodium falciparum*

**DOI:** 10.1186/s12936-022-04330-3

**Published:** 2022-10-27

**Authors:** Tanyaluck Kampoun, Somdet Srichairatanakool, Parichat Prommana, Philip J. Shaw, Judith L. Green, Ellen Knuepfer, Anthony A. Holder, Chairat Uthaipibull

**Affiliations:** 1 Department of Biochemistry, Faculty of Medicine, Chiang Mai University, Chiang Mai, 50200 Thailand; 2 Medical Molecular Biotechnology Research Group, National Center for Genetic Engineering and Biotechnology (BIOTEC), 113 Thailand Science Park, Phahonyothin Road, Khlong Nueng, Khlong Luang, Pathum Thani, 12120 Thailand; 3 Malaria Parasitology Laboratory, The Francis Crick Institute, 1 Midland Road, NW1 1AT London, UK; 4 Present Address: Molecular and Cellular Parasitology Laboratory, Department of Pathobiology and Population Sciences, The Royal Veterinary College, Hawkshead Lane, AL9 7TA Hatfield, UK; 5 Present Address: Thailand Center of Excellence for Life Sciences (TCELS), Phayathai, Bangkok, 10400 Thailand

## Abstract

**Background:**

The resistance of *Plasmodium falciparum* to artemisinin-based (ART) drugs, the front-line drug family used in artemisinin-based combination therapy (ACT) for treatment of malaria, is of great concern. Mutations in the *kelch13* (*k13*) gene (for example, those resulting in the Cys580Tyr [C580Y] variant) were identified as genetic markers for ART-resistant parasites, which suggests they are associated with resistance mechanisms. However, not all resistant parasites contain a *k13* mutation, and clearly greater understanding of resistance mechanisms is required. A genome-wide association study (GWAS) found single nucleotide polymorphisms associated with ART-resistance in *fd* (ferredoxin), *arps10* (apicoplast ribosomal protein S10), *mdr2* (multidrug resistance protein 2), and *crt* (chloroquine resistance transporter), in addition to *k13* gene mutations, suggesting that these alleles contribute to the resistance phenotype. The importance of the FD and ARPS10 variants in ART resistance was then studied since both proteins likely function in the apicoplast, which is a location distinct from that of K13.

**Methods:**

The reported mutations were introduced, together with a mutation to produce the *k13*-C580Y variant into the ART-sensitive 3D7 parasite line and the effect on ART-susceptibility using the 0−3 h ring survival assay (RSA_0−3 h_) was investigated.

**Results and conclusion:**

Introducing both *fd*-D193Y and *arps10*-V127M into a *k13*-C580Y-containing parasite, but not a wild-type *k13* parasite, increased survival of the parasite in the RSA_0−3 h_. The results suggest epistasis of *arps10* and *k13*, with *arps10*-V127M a modifier of ART susceptibility in different *k13* allele backgrounds.

**Supplementary Information:**

The online version contains supplementary material available at 10.1186/s12936-022-04330-3.

## Background

Artemisinin (ART)-based combination therapy (ACT) for *Plasmodium falciparum* malaria is the frontline drug combination recommended by the World Health Organization (WHO). ART and derivatives (ARTs) are the most effective drugs for parasite elimination since they are fast-acting, but they have a short half-life. ACT uses a treatment of ARTs with a second drug that has a longer half-life to ensure removal of all parasites. The emergence of parasite resistance to ARTs is extremely concerning. ART-resistance is characterized by slow parasite clearance in patients, and reduced susceptibility of ring-stage parasites in an in vitro 0−3 h ring survival assay (RSA_0−3 h_) [1]. Clinically resistant parasites were first detected in western Cambodia and have spread throughout the Greater Mekong Sub-region and beyond [2–4].

Mutations in the gene encoding the propeller domain of the Kelch13 (K13) protein were identified as genetic markers associated with ART-resistance [5]. The role of K13 in ART resistance has been clearly established by both forward and reverse genetic studies, but its biochemical function remains unclear. The level of resistance appears to be modulated by mutations in other genes that occurred during in vitro selection, suggesting that resistance may be multifactorial. For example, it was reported that a mutation in the PF3D7_0110400 gene occurred at the same time as the K13 mutation and that this was associated with resistance level in RSA_0−3 h_. However, mutations in PF3D7_0110400 have not been identified in resistant field isolates, suggesting that this mutation selected under in vitro drug pressure may not occur in nature. Another important question that arose from this study is whether mutations in other genes are epistatic to *k13* mutations, i.e., does the ART-resistance phenotype mediated by a *k13* allele depend on mutations in other genes?

A genome-wide association study (GWAS) of field-isolates reported *k13* alleles and single nucleotide polymorphisms (SNPs) at different loci associated with resistant founder populations in which *k13* mutations had arisen [6]. The reported SNPs were in *fd* (ferredoxin, *fd*-D193Y), *arps10* (apicoplast ribosomal protein S10, *arps10*-V127M), *mdr2* (multidrug resistance protein 2, *mdr2*-T484I), and *crt* (chloroquine resistance transporter, *crt*-N326S). The *fd*-D193Y SNP (Asp193Tyr substitution in ferredoxin) was most strongly associated with resistant founder populations and the *arps10*-V127M SNP (Val127Met substitution in apicoplast ribosomal protein S10) was the SNP most strongly associated with delayed parasite clearance after *k13*-C580Y, the most common of the *k13* alleles found in the Greater Mekong Subregion (GMS) [7]. Hence, the questions raised by this GWAS study include: do the reported SNPs, *fd*-D193Y and *arps10*-V127M contribute to ART resistance, and is there an epistatic relationship between the *k13* mutant and the reported SNPs in an ART-resistance phenotype? Both FD and ARPS10 are nuclear encoded apicoplast proteins, therefore, do different alleles of these two genes affect the function of the proteins and of the apicoplast, to provide an advantage to the parasite for survival under drug pressure? Apicoplast function includes type II fatty acid biosynthesis, *de novo* haem biosynthesis, and isoprenoid biosynthesis, but only isoprenoid biosynthesis is thought to be important in the asexual blood stage of the life cycle [8].

To examine the effect of the SNPs identified in the GWAS study, and genetic epistasis with the *k13* allele, genome editing was performed to introduce specific mutations into the target genes. In an earlier genome editing study, *k13* mutations from Cambodian field isolate parasites were reported to confer ART resistance [9]. Editing the wild type *k13* gene in the ART-sensitive parasite to introduce the reported *k13* mutations significantly increased the survival of the parasite in RSA_0−3 h_, and converting *k13* mutants to *k13* wild type sequence in ART-resistant parasite lines from Cambodian isolates restored parasite sensitivity to ART. The introduction of different *k13* mutations into the same parasite line resulted in differences in the parasite survival and the introduction of the same *k13* mutation into different parasite lines also resulted in differences in the parasite survival, indicating that the parasite’s genetic background affects the level of resistance conferred by individual *k13* mutations.

The CRISPR/Cas9 genome editing system was used to modify the ART-sensitive 3D7 parasite line. Mutations to produce *k13-*C580Y, *fd*-D193Y and *arps10*-V127M either individually or together was performed and 3D7 was used for the experiment control, so the genetic background of all modified parasites was the same. 3D7 is a laboratory *P. falciparum* line originally from Africa where the spread of ART resistance is concerning. The *fd* and *arps10* mutations were expected to modulate sensitivity to ART, but only in a *k13* mutant background so the *fd* and *arps10* mutations were introduced into 3D7 and a *k13*-C580Y containing 3D7.

## Methods

### *Plasmodium falciparum* culture and synchronization

Parasites were cultured in a complete RPMI 1640 medium (RPMI 1640 medium with 2 mM l-glutamine, 25 mM HEPES, 2 g/L NaHCO_3_, 27.2 mg/L hypoxanthine and 0.5% Albumax II, pH7.4) using human O + erythrocytes (2−4% haematocrit). Parasite cultures were gassed with 90% N_2_, 5% CO_2_ and 5% O_2_ and incubated at 37 °C. Parasite developmental stage and viability were routinely assessed by microscopic examination of Giemsa-stained thin blood films. Before transfection, parasite populations were tightly synchronized using a 70% Percoll gradient to purify schizont stages. Synchronous ring stage parasite populations were produced from purified schizonts by allowing merozoite invasion for three hours, followed by 5% D-sorbitol treatment to remove residual schizonts as described in [10].

### Plasmid design and construction for mutagenesis

The genomic DNA sequences of *Pfk13* (PF3D7_1343700), *Pffd* (PF3D7_1318100) and *Pfarps10* (PF3D7_1460900.1) were obtained from PlasmoDB, Plasmodium Informatics Resources (www.PlasmoDB.org) and used for plasmid, oligonucleotide, and primer design.

To construct Cas9 plasmids, three independent guides for the gene of interest (GOI) were designed to direct Cas9 cutting to near the codon to be modified. A pair of complementary oligonucleotides were phosphorylated, annealed, and cloned into the pDC2-cam-Cas9-U6-hDHFRyFCU-plasmid [10] via a *Bbs*I restriction site for pCas9_pfk13g, pCas9_pffdg and pCas9_pfarps10g, used for mutagenesis of codons to produce the *k13*-C580Y, *fd*-D193Y and *arps10*-V127M variants, respectively. The pairs of complementary oligonucleotides are shown in Additional file 1: Table S1.

To construct a repair plasmid to introduce the required mutation, a recodonized sequence was designed, containing the modified codon and flanked with homologous regions (HR) for integration into the target GOI. The recodonized region of the guide RNA encoded the same amino acids as the endogenous sequence except for the codon to be changed and changes to prevent Cas9 cleavage of the modified sequence. For the repair plasmid to generate *k13*-C580Y (pMA_k13MT), a 42 bp recodonized sequence region was flanked with pfk13HR1 (310 bp) and pfk13HR2 (360 bp), respectively. The DNA sequence was synthesized and cloned into pMA, an ampicillin resistance plasmid, by GeneArt, Life Technologies. For the repair plasmid to generate *fd*-D193Y (pMK_fdMT), a 40 bp recodonized sequence was flanked with pffdHR1 (400 bp) and pffdHR2 (406 bp), respectively. The DNA sequence was synthesized by GeneArt and provided in pMK, a kanamycin resistance plasmid. For the repair plasmid to generate *arps10*-V127M (pGEM-T_arps10MT), a 21 bp recodonized sequence region was flanked with pfarps10HR1 (339 bp) and pfarps10HR2 (346 bp), respectively. To construct this plasmid, pairs of primers were designed to amplify pfarps10HR1 and pfarps10HR2 from 3D7 genomic DNA. The reverse primer for pfarps10HR1 (arps10HR1_R) and the forward primer for pfarps10HR2 (arps10HR2_F) were designed to amplify overlapping sequences with the generation of the recodonized sequence region between pfarps10HR1 and pfarps10HR2. Following PCR amplification of pfarps10HR1 and HR2, the products were used in a second PCR reaction using arps10HR1_F and arps10HR2_R to generate the full DNA repair sequence, which was cloned into the pGEM-T Easy vector. The plasmid structure was confirmed by restriction enzyme digestion and sequence analysis. Repair plasmid sequences are provided in Additional file 2: Table S2.

### ***Plasmodium falciparum*** transfection and cloning

**Fig. 1:**
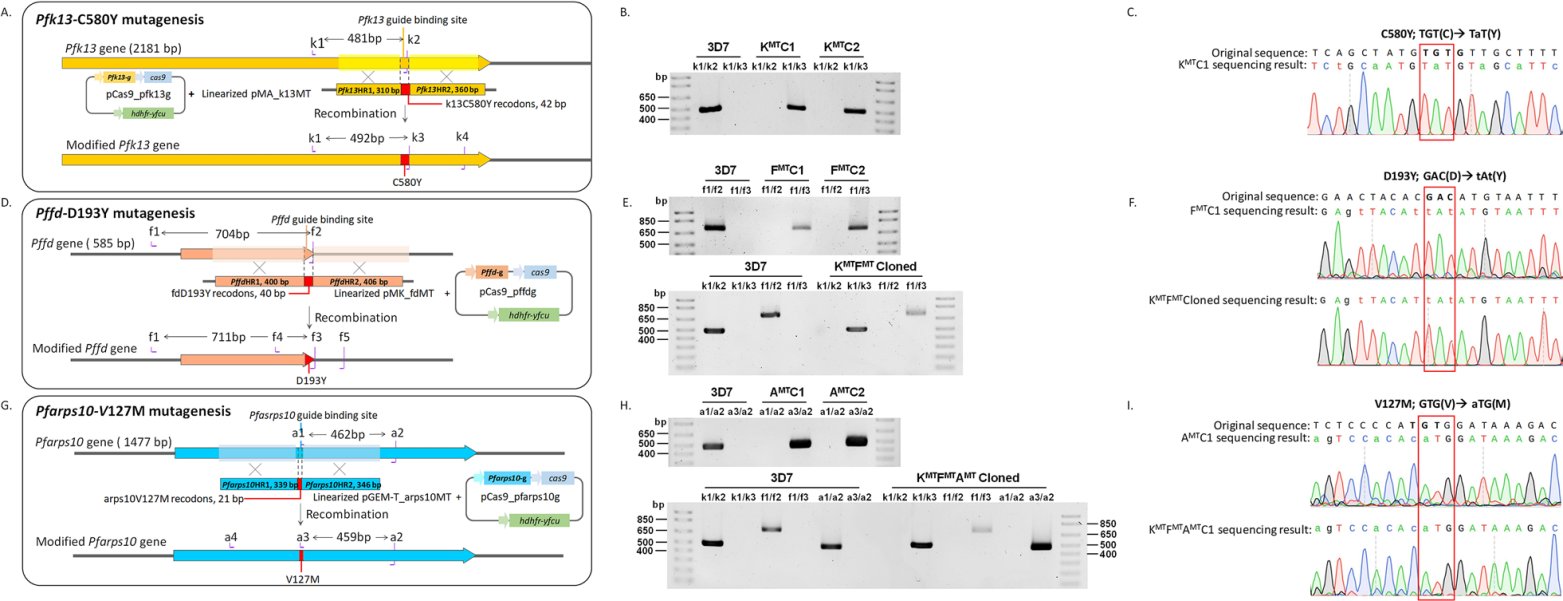
Strategy to introduce the *k13*-C580Y, *fd****-***D193Y and *arps10*-V127M alleles.** A** *k13*-C580Y mutagenesis. Plasmid pCas9_pfk13g and linearized plasmid pMA_k13MT were co-transfected into the 3D7 parasite; pCas9_pfk13g contains expression cassettes for Cas9 and k13 specific guide RNA for generating a double-strand break in k13. The *k13*-C580Y mutation was introduced by homology-directed DNA repair using k13HR1 and k13HR2 sequences in pMA_k13MT. The k1/k2 primer pair was used to identify the wild-type genotype (product size = 481 bp) of the unmodified parasite and the k1/k3 pair was used to identify the modified gene (product size = 492 bp). **B** Genotype analysis by PCR amplification of the modified parasite after cloning. Two transgenic parasite clones (K^MT^C1 and K^MT^C2) were isolated and tested by PCR analysis in comparison with 3D7 (**C**) DNA sequence analysis of K^MT^C1. The *k13*-C580Y mutated codon is highlighted in red (TGT(C) ◊ TaT(Y)). **D** *fd*-D193Y mutagenesis. The pCas9_pffd and pMK_fdMT plasmid pair was used for *Pffd* gene modification. The f1/f2 and f1/f3 primer pairs were used to identify unmodified parasites (product size = 704 bp) and modified parasites (product size = 711 bp), respectively. For the sequence analysis, the f4/f5 primer pair was used to amplify and read the sequence. **E** The genotype analysis of the *fd*-D193Y mutated parasites. **E** (upper) PCR result for clonal parasites (F^MT^C1 and F^MT^C2) with single *fd*-D193Y mutation. **E** (lower) PCR result for cloned parasite (K^MT^F^MT^) with double mutation k13^C580Y^fd^D193Y^. **F** DNA sequence analysis result confirming the *fd*-D193Y mutation: the sequence result for the F^MT^C1 parasite (**F**, upper) and K^MT^F^MT^ (**F**, lower) with the *fd*-D193Y mutated codon highlighted in red (GAC(D) ◊ tAt(Y)). **G** *arps10*-V127M mutagenesis. The pCas9_pfarps10 and linearized pGemT_arps10MT plasmids were used to modify the *arps10* gene in 3D7 and K^MT^F^MT^ parasites. The a1/a2 (product size = 462 bp) and a3/a2 (product size = 459 bp) primer pairs were used to identify unmodified parasite and modified parasite, respectively. The a4/a2 primer pairs were used to amplify and read the sequence for sequence analysis. **H** PCR genotypic analysis: (**H**, upper) PCR result for two clonal lines of A^MT^. (**H**, lower) PCR result for one clonal line of K^MT^F^MT^A^MT^. **I** DNA sequence analysis confirming *arps10*-V127M mutation. Data are shown for A^MT^ (**I**, upper) and K^MT^F^MT^A^MT^ (**I**, lower) clonal lines with the *arps10*-V127M mutated codon highlighted in red (GTG(V) ◊ aTG(M)). *Pfk13*-g, *Pffd*-g or *Pfarps1*0-g: *Pfk13*, *Pffd* or *Pfarps10* specific guide RNA gene cassette, *cas9*: Cas9 gene cassette, hdhfr-yfcu: drug selectable marker, HR; Homologous region, K^MT^ = k13^C580Y^ mutation, F^MT^ = fd^D193Y^ mutation, A^MT^ = arps10^V127M^ mutation

Sixty µg of linearized repair plasmid and 20 µg of CRISPR/Cas9 plasmid carrying the specific guide RNA sequence were co-precipitated with ethanol and then dissolved in 10 µl sterile TE (10 mM Tris-HCl 1 mM EDTA pH 8.0). This transfection DNA mixture was added to 100 µl of AMAXA primary cell solution P3, mixed with 10 to 40 µl of synchronized mature schizonts and electroporated using an Amaxa 4D-Nucleofector™ (Lonza) with program FP158. To select for parasites with the Cas9/guide plasmid, the transfected parasites were cultured in 2.5 nM WR99210 for four days. Then, the cultures were treated with 1 µM 5-fluorocytosine (5-FC) for one week to negatively select parasites retaining the Cas9/guide plasmid. The parasite population was screened for integration by PCR analysis, using the pairs of primers shown in Fig. 1A, D, G. Then, the transgenic parasites were cloned by limiting dilution and screening by PCR. The presence of the mutant sequence in cloned parasites was confirmed by DNA sequence analysis of PCR products, using the primers described in Fig. 1A, D, G.

### Parasite growth assay

Growth analysis of the parental 3D7 and modified parasites was performed over two intraerythrocytic replication cycles. Tightly synchronized ring stage parasite populations (0−3 h post-invasion) were prepared at 0.2% parasitaemia and 2% haematocrit. Parasite morphology and percentage parasitaemia were determined by microscopic examination of Giemsa-stained blood films at 0 h, 24 h, 48 h, 72 and 96 h time points to assess parasite growth and development. Experiments were performed three times, each with two technical replicates. Data for each modified parasite were compared to those for parental 3D7 parasites and analysed statistically using a one-way ANOVA analysis with Dunnett’s multiple comparisons test.

### ART-susceptibility assessment by 0−3 h ring survival assay (RSA_0−3 h_)

Synchronous populations of mature schizonts were prepared by Percoll-purification and released merozoites were allowed to invade new red blood cells for exactly 3 h (producing 0−3 h ring stages), followed by 5% sorbitol treatment to remove residual schizonts. The culture was prepared at 0.5−1% parasitaemia and 2% haematocrit, and Giemsa-stained thin smears were prepared to measure the initial percent parasitaemia (INI). Cultures were treated with either 0.1% DMSO (non-DHA exposed control [NE]), 20 nM DHA or 700 nM DHA for 6 h in 48-well plates. Each parasite sample was then washed once with 9 ml RPMI 1640, resuspended in 1 ml complete medium, placed in a new well, and cultured for a further 66 h. Giemsa stained smears were prepared for microscopic analysis of parasite morphology and percent parasitaemia, in which at least 10,000 erythrocytes were counted for each sample. Assays were repeated 5 or 6 times independently, with two technical repeats for each sample. Growth and survival were calculated by the formulae below. Only if the growth was ≥ 1.5 was the percent survival rate calculated.

$$\text{Growth} =\frac{{\%}\, {\text{of NE}}}{{\%}\,\text{of}\,\text{INI}}, \quad {\%}\, \text{Survival}=\frac{{\%} {\text{ of DHA exposed parasite}}}{{\%}\, {\text{of NE} }} \times 100.$$

GraphPad Prism 8.3.0 was used for all statistical analyses. One-way ANOVA with Tukey’s multiple comparisons test was used to compare the mean survival of each parasite to the mean survival of every other. One-way ANOVA with Dunnett’s multiple comparisons test was used to compare the mean growth or the mean percentage of the growth stages of each parasite to the corresponding mean of the control parasite. P < 0.05 was considered statistically significant.

## Results

### Transgenic parasites established by CRISPR/Cas9 mutagenesis

Plasmids were designed and prepared for mutagenesis to insert the *k13*-C580Y, *fd*-D193Y and *arps10*-V127M mutations into the 3D7 parasite genome. For each mutagenesis, a Cas9 and a repair plasmid were designed and constructed (Additional files 1, 2: Tables S1, S2), co-transfection was performed, and then modified parasites were screened, selected and cloned (Fig. 1).

To insert the *k13*-C580Y mutation, following transfection and selection the modified parasites were examined using PCR with primer pairs k1/k2, designed to amplify only wildtype unmodified *k13* sequence and the primer pairs k1/k3, designed to amplify only modified *k13* sequence (Fig. 1A). PCR analysis of two parasite lines, selected and cloned by limiting dilution, revealed a positive product only with the k1/k3 primer pair while the parental 3D7 parasite was only positive with the k1/k2 primer pair. This indicates that the transgenic parasites were successfully modified and contain only the *k13*-C580Y allele (Fig. 1B). DNA sequence analysis of the region of *k13* amplified using the k1/k4 primer pair confirmed that the desired mutation had been introduced into the parasite (Fig. 1C), and this clone was used in subsequent experiments.

To investigate the contribution of *fd*-D193Y to ART resistance, the *fd*-D193Y mutation was introduced into both the parental 3D7 and transgenic k13^C580Y^_3D7 parasite. Modified parasites were screened by PCR analysis: the f1/f2 primer pair was designed to amplify wildtype-*fd* and the f1/f3 primer pair was designed to specifically amplify the modified-*fd* sequence (Fig. 1D). Two parasite clones containing the modified *fd* allele were confirmed by PCR analysis: they were positive only with the f1/f3 primer pair, and contained the *fd*-D193Y allele (Fig. 1E, upper). Following mutagenesis of the k13^C580Y^_3D7 parasite to introduce the *fd*-D193Y allele, the transgenic parasite was selected, cloned and the sequence confirmed using the same strategy. The *fd*-modified k13^C580Y^_3D7 was positive only in a PCR reaction with the f1/f3 primer pair, so the transgenic parasite was k13^C580Y^fd^D193Y^_3D7 (Fig. 1E, lower). The DNA sequence analyses confirmed that the *fd* gene of 3D7 (Fig. 1 F, upper) and k13^C580Y^_3D7 (Fig. 1F, lower) parasites had been modified to *fd*-D193Y. The confirmed fd^D193Y^_3D7 and k13^C580Y^fd^D193Y^_3D7 parasite lines were used in subsequent experiments.

The ART-resistant parasite from a field isolate sampled in the GWAS contained multiple SNPs in addition to those in *k13* and *fd*, a further modification to the k13^C580Y^fd^D193Y^_3D7 parasite was performed. Of particular interest was the *arps10*-V127M allele, the k13^C580Y^fd^D193Y^ arps10^V127M^_3D7 parasite, and also arps10^V127M^_3D7 were established to observe any effect of *arps10*-V127M alone on ART resistance. The *arps10*-V127M mutagenesis was performed on the 3D7 and k13^C580Y^ fd^D193Y^_3D7 parasite lines and the products analysed by PCR, using the primers shown in Fig. 1G. Two clones of *arps10*-modified 3D7 parasites were confirmed by PCR analysis to have the *arps10*-V127M allele, with 3D7 used as a control for comparison. Both selected clones were *arps10*-modified parasites (Fig. 1H, upper). For *arps10*-modified k13^C580Y^ fd^D193Y^_3D7, the selected clone was confirmed to contain the *k13*-C580Y, *fd*-D193Y and *arps10*-V127M alleles by PCR analysis, so the *arps10*-modified k13^C580Y^ fd^D193Y^_3D7 had been modified and still carried the *k13*-C580Y and *fd*-D193Y alleles (Fig. 1H, lower). DNA sequence analysis confirmed the presence of the *arps10*-V127M allele in *arps10*-modified 3D7 (Fig. 1I, upper) and *arps10*-modified k13^C580Y^fd^D193Y^_3D7 (Fig. 1I, lower) parasites lines.

### Growth of the transgenic parasites was the same as that of the 3D7 parasite

**Fig. 2:**
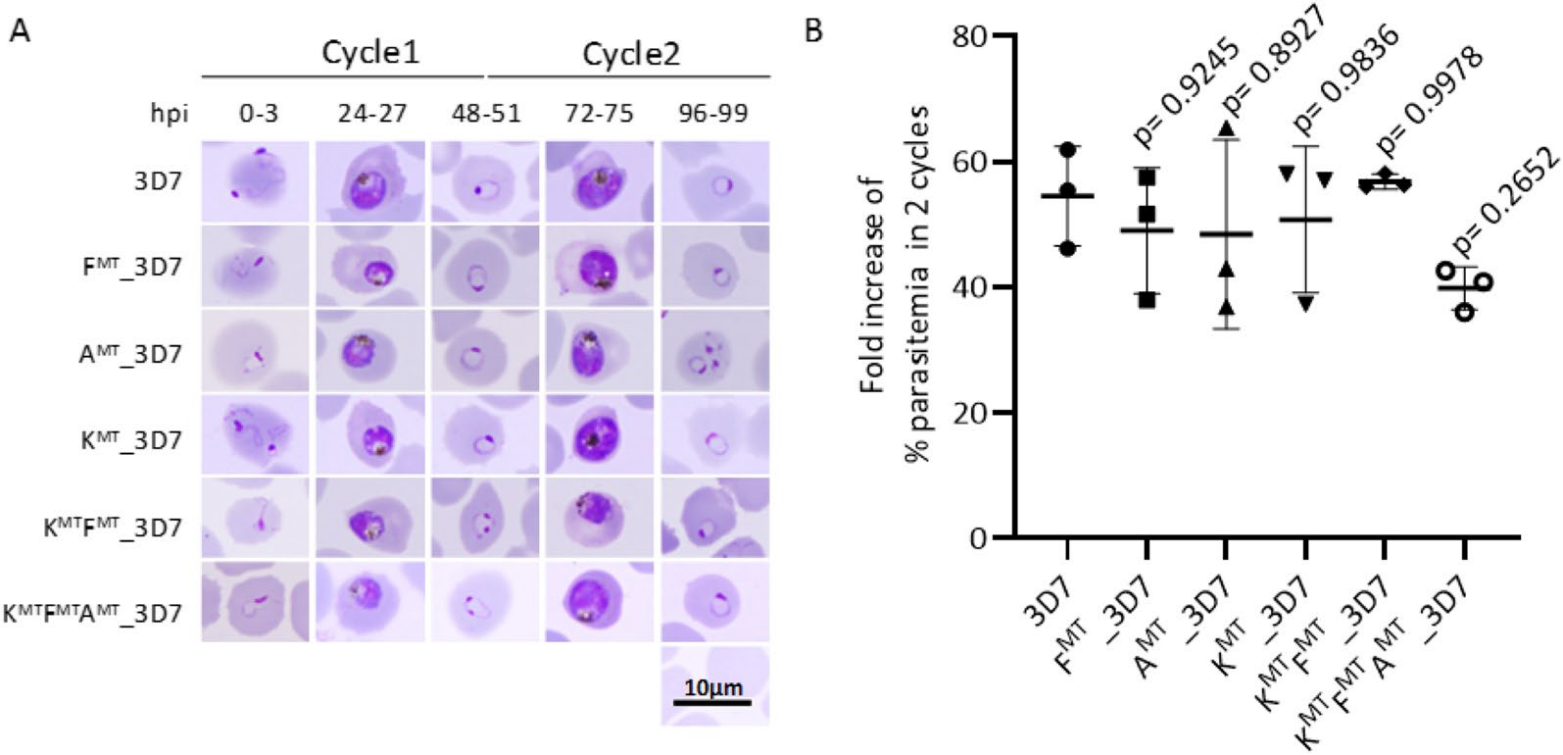
Growth and development of the modified parasites. **A** Giemsa-stained parasites at 0, 24, 48, 72 and 96 h after initial invasion (hpi). Most parasites in each population of modified parasites were at the same stage of development as parental 3D7 parasites. **B** Fold increase in percentage parasitaemia over 2 cycles (96 h). The results are shown as means ± SD of 3 independent experiments. One-way ANOVA with Dunnett’s multiple comparisons test was performed. The growth of all transgenic parasites was not significantly different from 3D7 (95% CI). K^MT^ = k13^C580Y^ mutation, F^MT^ = fd^D193Y^ mutation, A^MT^ = arps10^V127M^ mutation

To examine the effect of each mutation on parasite growth, transgenic parasites were compared with the parental 3D7 parasite line over two erythrocytic development cycles. No differences in morphology were apparent among transgenic lines compared with 3D7 at each time point, suggesting that development was unaffected by the mutations (Fig. 2A). At the end of cycle 2, the fold increase of all transgenic parasites was not significantly different from that of 3D7 (one-way ANOVA with Dunnett’s multiple comparisons test, three independent experiments) (Fig. 2B). In conclusion, the alleles *k13*-C580Y, *fd*-D193Y and *arps10*-V127M alone, *k13*-C580Y together with *fd*-D193Y, or *k13*-C580Y, *fd*-D193Y and *arps10*-V127M together, did not significantly affect parasite proliferation or development compared with 3D7, suggesting that there is no substantial fitness cost associated with these alleles in vitro.

### Phenotypic analysis of parasite ART-resistance by ring stage survival assay

The susceptibility of the modified parasites to ART was examined using the in vitro 0 to 3 h ring survival assay (RSA_0 − 3 h_) to determine percent survival after a 6 h treatment with 700 nM DHA [11]. The lower 20 nM DHA concentration was also tested to further differentiate ART-sensitive from ART-resistant parasites [12]. The 3D7 parental line was used as the ART-sensitive parasite control and MRA1240 (IPC 5202) was used as an ART-resistant parasite control; MRA1240 is a parasite line from a malaria patient in western Cambodia containing *k13*-R539T, which confers high resistance to ART in RSA_0 − 3 h_. Growth was also measured for untreated parasites over the same period as RSA_0−3 h,_ providing controls to interpret the percentage survival values (the parasite growth must be ≥ 1.5).

**Fig. 3:**
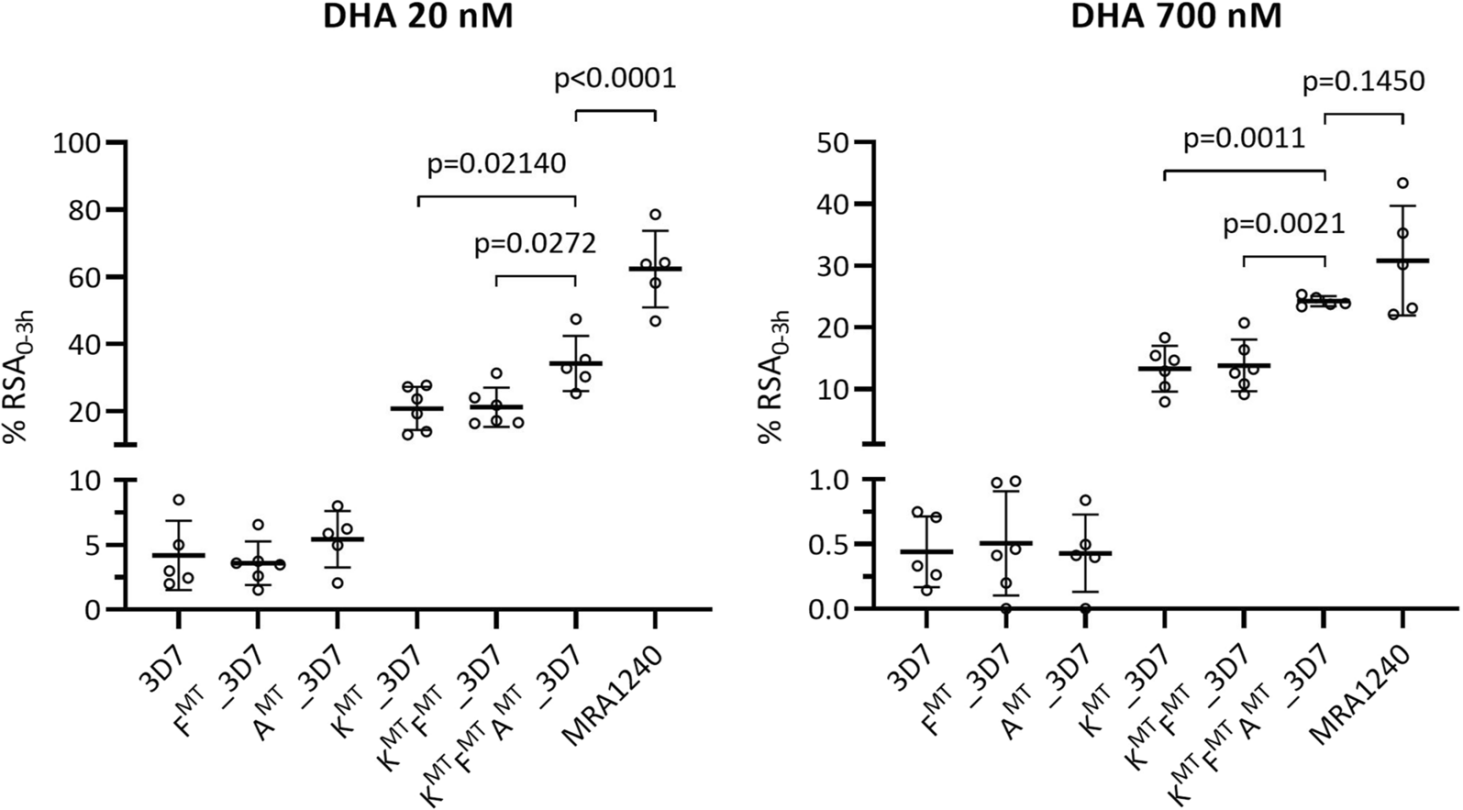
Percentage of parasite ring survival in RSA _0−3 h_. Univariate scatterplots of the % survival for 20 nM DHA treatment (left) and 700 nM DHA treatment (right) for the modified parasite lines, parental 3D7 and ART-resistance control strain (MRA1240). Mean and error bars (SD) are also shown for each sample group. One-way ANOVA with Tukey’s multiple comparisons test was performed. The dotted line at 1% survival shows the cut off used to discriminate between sensitive and resistant lines in the 700 nM DHA treatment. K^MT^ = k13^C580Y^ mutation, F^MT^ = fd^D193Y^ mutation, A^MT^ = arps10^V127M^ mutation

As expected, the survival of 3D7 following 700 nM DHA treatment was below 1%, whereas that of MRA1240 was greater than 30%. The transgenic parasites with the K13^C580Y^ mutation (i.e. the K^MT^, K^MT^F^MT^, and K^MT^F^MT^A^MT^ parasite lines) also showed greater than 1% survival, and therefore they are also defined as ART-resistant (Fig. 3; Table 1). The survival of F^MT^ in 20 nM and 700 nM DHA was not significantly different to the survival of 3D7 in both DHA treatments, unlike the increased survival of K^MT^. The survival of K^MT^F^MT^ in 20 nM DHA and 700 nM DHA was not significantly different from the corresponding survival of K^MT^. Therefore, the *fd*-D193Y allele in either the 3D7 or the K13^C580Y^ parasite lines did not affect the parasite’s ART-sensitivity phenotype. The survival of A^MT^ in 20 nM and 700 nM DHA was not significantly different from the corresponding survival of 3D7. The survival of the triple mutant K^MT^F^MT^A^MT^ in 20 nM and 700 nM DHA was significantly different from the corresponding survival of the single (K^MT^) and double mutant (K^MT^F^MT^) in 20 nM and 700 nM DHA. Therefore, the ARPS10^V127M^ mutation appears to modulate ART sensitivity depending on the genetic background. Interestingly, the survival of K^MT^F^MT^A^MT^ was not significantly different from that of MRA1240 in 700 nM DHA, wherease in 20 nM DHA the survival differed.

**Table 1:** The growth and % survival results in RSA_0−3 h_ assay of transgenic parasite lines, their parental strain and control of ART-resistance parasite (mean ± SD)

Parasite sample	Growth	P value	RSA_0–3 h_ (%), 20 nM DHA	P value	RSA_0–3 h_ (%), 700 nM DHA	P value
3D7	5.25 ± 1.63		4.19 ± 2.67		0.44 ± 0.27	
F ^MT^ _3D7	5.08 ± 0.94	> 0.9999	3.58 ± 1.68	> 0.9999	0.51 ± 0.40	> 0.9999
A ^MT^_3D7	3.89 ± 1.01	0.7859	5.42 ± 2.18	> 0.9999	0.43 ± 0.30	> 0.9999
K ^MT^ _3D7	3.57 ± 0.84	0.5384	20.88 ± 6.42	0.0023*	13.26 ± 3.73	0.0001**
K ^MT^ F ^MT^ _3D7	4.70 ± 1.24	0.9964	21.25 ± 5.87	0.0017*	13.80 ± 4.18	< 0.0001***
K ^MT^ F ^MT^ A ^MT^ _3D7	4.27 ± 0.68	0.9442	34.29 ± 8.26	< 0.0001**	24.23 ± 0.83	< 0.0001***
MRA1240	5.99 ± 3.14	0.9864	62.36 ± 11.46	< 0.0001**	30.80 ± 8.88	< 0.0001***

Each experiment was repeated for 5–6 times independently, with each group comprised of two technical repetitions. One-way ANOVA with Tukey’s multiple comparisons test was performed at 95% CI. The P-values of the comparison of each strain to the 3D7 line are showed

### Effect of DHA treatment on parasite growth and morphology

**Fig. 4:**
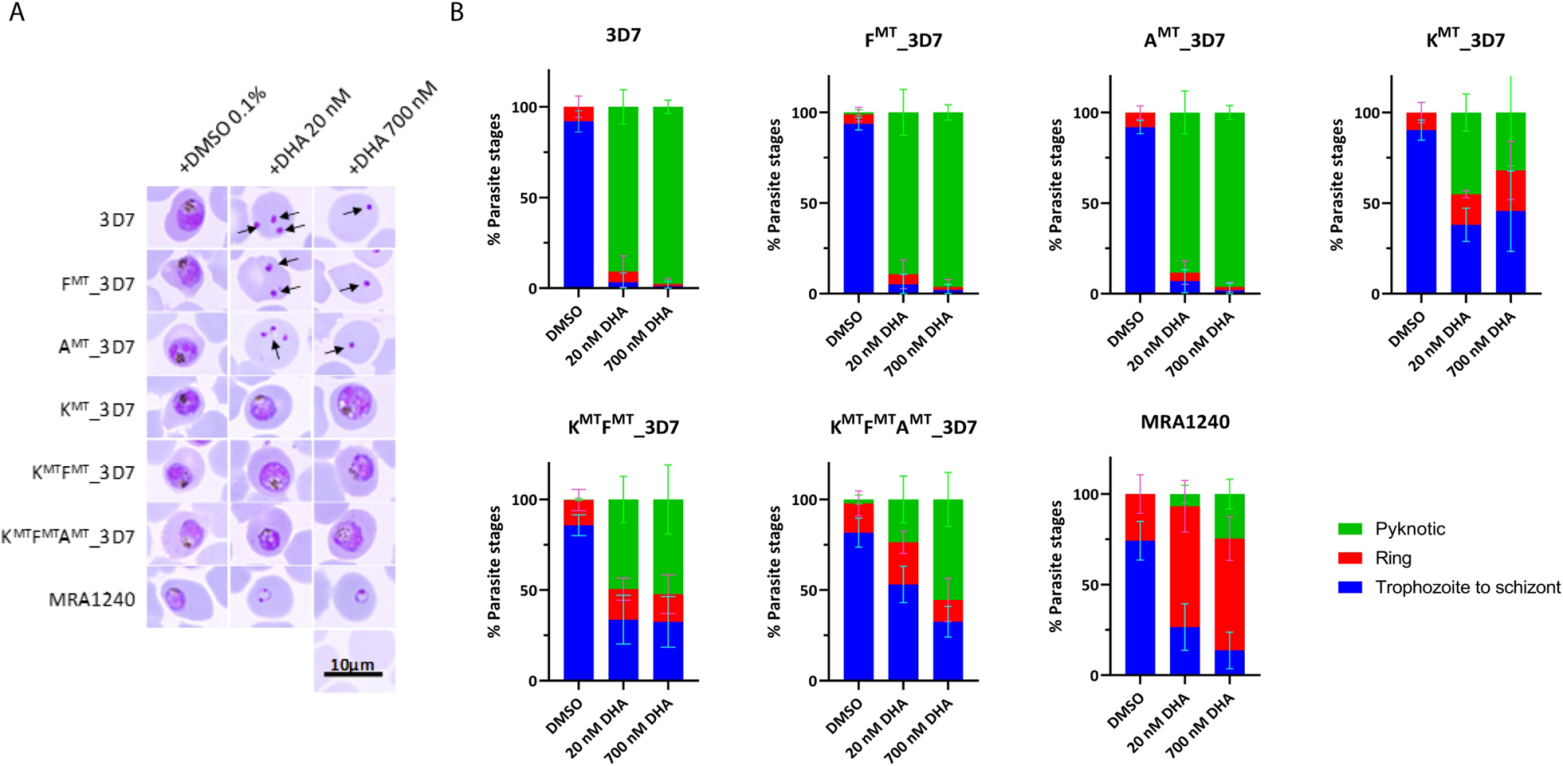
Parasite morphology in control (mock treatment; + 0.1% DMSO) or drug treated (+ 20 nM DHA and + 700 nM DHA) cultures. **A** Representative images from Giemsa-stained thin smears showing morphology of parasites at 72 h of treatment. Most of the 3D7, F^MT^ and A^MT^ parasites treated with DHA were identified as pyknotic and dead (indicated by arrows). In contrast, a marked proportion of K^MT^, K^MT^F^MT^, and K^MT^F^MT^A^MT^ parasites appeared viable and mostly at the late trophozoite and early schizont stages of development. MRA1240 parasites also appeared viable and mostly at the ring stage, with some early trophozoites, consistent with some growth delay. **B** The percent infected red cells containing trophozoites and schizonts (blue), rings (red) and pyknotic forms (green)

The effect of DHA treatment on parasite growth and morphology was assessed by light microscopy (Fig. 4). At 72 h following mock treatment, in all samples most parasites were viable and at late trophozoite to early schizont stages of development. Following DHA treatment, most 3D7, F^MT^ and A^MT^ parasites were identified as pyknotic and dead (indicated by arrows). In contrast, a much greater proportion of K^MT^, K^MT^F^MT^, and K^MT^F^MT^A^MT^ parasites appeared to be viable late trophozoite and early schizont stages. MRA1240 parasites also appeared viable and mostly at the ring stage, with some early trophozoites, consistent with growth delay. The percent of trophozoite/schizont and ring stage parasites in each parasite and treatment group was compared statistically using one-way ANOVA, and only MRA1240 was significantly different in the distribution of parasite stages when compared with 3D7 (Additional file 3: Fig. S1).

## Discussion

Most ART-resistant parasites from field isolates contain *k13* mutations. Several different *k13* alleles have been identified and associated with different levels of ART resistance. In addition to the *k13* alleles, SNPs in other genes have been identified that are associated with ART resistance, and it is important to establish whether or not these genes have a direct or indirect role in the manifestation of the resistance phenotype. If many genetic factors drive resistance, then it will be important to establish the role of specific genes and individual alleles of those genes. GWAS studies have identified a high prevalence of SNPs associated with resistance, and it is possible that *k13* alleles and different alleles of other genes are epistatic in modulating parasite survival in the presence of artemisinin. Therefore, it is of interest to establish whether, within a defined genetic background and with a single *k13* allele, mutations in other genes may modify parasite survival in the presence of ART.

The mutations of particular interest were *fd*-D193Y and *arps10*-V127M, which were reported to be highly associated with ART-resistance in a GWAS. Ferredoxin is a protein that functions in the apicoplast and the *fd*-D193Y allele has been implicated in ART-resistance. *Plasmodium falciparum* ferrodoxin (*Pf*Fd) interacts with *P. falciparum* ferredoxin NADP^+^ reductase (*Pf*FNR) in a major redox system of the apicoplast. Fd-FNR interaction studies suggest that Fd-D193Y (D97Y at the C-terminus of mature ferredoxin in the apicoplast) is within an important interaction site for electron exchange. Site-directed mutagenesis to substitute *Pf*Fd C-terminal residues indicated that an aromatic residue at positions 96 and 97 increased the *Pf*Fd-*Pf*FNR binding affinity. A recent study analysing the interaction of *Pf*Fd-D193Y and *Pf*FNR showed mutations leading to attenuation of *Pf*FNR function and suggested that this could be associated with the action of artemisinin [13, 14]. ARPS10 has a role in apicoplast protein translation. *k13* mutations have been reported to deregulate phosphorylation of *Pf*PK4, leading to downstream phosphorylation of eukaryotic translation initiation resulting in reduced rate of intraerythrocytic development via regulation of translation. This leads to an extended ring stage and lower haemoglobin catabolism, resulting in reduced levels of free Fe(II)PPIX available to activate artemisinin [15]. It is possible that *arps10* mutations might affect the level of apicoplast protein(s), which interact directly with the mechanism of ART-resistance or enhance the effect of *k13* mutation.

These hypotheses were investigated by measuring the RSA survival of parasites with *k13* wild-type or *k13* mutant alleles and *fd* and *arps10* alleles in the same genetic background. To clarify the potential interaction of *fd*-D193Y and *arps10*-V127, these mutant genes were introduced into a single laboratory sensitive parasite strain to remove other genetic factors that could interfere with the analysis. There are several studies that introduced the *k13*-C580Y allele into the 3D7 parasite line [12, 16–18]. The measured survival of the *k13*-C580Y-containing 3D7 from previous studies varied from 0.9 to 30.6%. The measured survival of K^MT^ was similar to that measured for 3D7 expressing K13^C580Y^-HA, for which the survival of the parasite was about 9% [16], validating the approach used and the generated transgenic ART-resistant parasites in this study.

The first finding in this study was that none of the genetic modifications had any apparent deleterious effect on parasite growth in the absence of drug. This indicates that there might be no fitness cost associated with these mutations in the asexual blood stages of the life cycle. This is important because a mutation that enhances ART-resistance but causes the parasite to grow less well in the absence of drug is unlikely to become fixed in the population. A previous study reported a growth defect in their transgenic 3D7^C580Y^parasite, determining the growth defect by co-culture of the parasite line with 3D7^WT^. They compared the in vitro growth rates for 36 days to quantify the proportion of each *k13* allele, while in our experiment the growth was only examined for 4 days.

The addition of *fd*-D193Y or *arps10*-V127M alone into 3D7 did not make parasites resistant to ART in the RSA_0 − 3 h_ since their survival in 700 nM DHA treatment was < 1%. However this does not mean that *fd*-D193Y or *arps10*-V127M do not have a role in the ART-resistance mechanism, if there is a possibility that their contribution depends on the *k13* allele present. Therefore, these mutations were introduced into a parasite that already partially resists ART and examined the effect on ART-susceptibility. No difference was observed in ART-susceptibility between K^MT^F^MT^ and K^MT^parasites. It is possible that the *fd*-D193Y mutation may have an insufficient effect on Fd function to cause a measurable difference in ART susceptibility. This result is in line with that of a recent study in which reversion of *fd*-D193Y to wild-type did not increase parasite susceptibility to ART [18]. However, addition of *arps10*-V127M into the K^MT^F^MT^ parasite did enhance the resistance level as shown by a 1.6–1.7 fold increase in the survival of K^MT^F^MT^A^MT^ compared with the K^MT^ and K^MT^F^MT^ parasite lines.

The survival of the K^MT^F^MT^A^MT^ line to that of MRA1240 was also compared. MRA1240 is an ART-resistant parasite isolated from a malaria patient in western Cambodia, and a representative of resistant parasites in nature. The *fd* and *arps10* genes of MRA1240 were checked by PCR amplification and sequence analysis; MRA1240 parasites contain the *fd*-D193Y and *arps10*-V127M alleles, respectively (Additional file 4: Fig. S2). This finding correlates with the GWAS report that *fd*-D193Y and *arps10*-V127M are genetic background markers of *k13* mutant-containing parasites, correlating with the ART resistant parasite population [6]. The survival of K^MT^F^MT^A^MT^ was no different from that of MRA1240 following 700 nM DHA treatment which was unexpected because MRA1240 contains the *k13*-R539T allele expected to confer a higher ART resistance than *k13*-C580Y. Interestingly, following 20 nM DHA treatment the K^MT^F^MT^A^MT^ parasite showed greater sensitivity than MRA1240; *k13*-R539T may lead to greater loss of K13 function in parasites and consequently greater ART resistance, although both R539T and C580Y have a similar effect on K13 folding in vitro [19]. Perhaps it also reflects the complexity of the mode of action of ART and the resistance mechanisms evolved by the parasite. Since both *fd*-D193Y and *arps10*-V127M alleles show enhanced survival with the *k13*-C580Y allele in RSA_0−3 h_, so differences in the *k13* allele background might produce different effects on ART-resistance. It is possible that the *fd*-D193Y and *arps10*-V127M alleles are important to MRA1240 survival in RSA_0 − 3 h_. The current assays for ART susceptibility of parasites may not be sensitive enough to detect small contributions to the overall phenotype and this may explain why a phenotypic effect of *fd*-D193Y could not observed. The finding in this study is consistent with the observed prevalence of *fd*-D193Y and *arps10*-V127M alleles in field isolates with high levels of ART resistance. Both *fd*-D193Y and, in particular, *arps10*-V127M alleles may be required to confer high-level ART resistance acting epistatically with different *k13* alleles and in diverse genetic backgrounds.

The Fd-FNR redox system is an important supplier of electrons in the apicoplast for isoprenoid precursor synthesis [20]. Defects in ferredoxin might affect isoprenoid synthesis leading to a decrease of precursors for molecules such as PI3P, important for vesicular formation. ARPS10 is important in apicoplast protein translation, for example synthesis of Suf B, encoded in the apicoplast genome, that is a part of the Suf BCD assembly complex in the Suf iron-sulfur cluster synthesis pathway (Suf pathway). A defect in ARPS10 might affect the translation of Suf B, which consequently affects the Suf pathway [21]. The Suf pathway provides essential FeS-cluster cofactors for enzymes in the isoprenoid synthesis pathway including Fd, IspH and IspG. Isoprenoid synthesis provides substrate for protein prenylation, for example prenylated proteins driving haemoglobin vesicular trafficking. Recently, it was proposed that a defect in vesicular trafficking is involved in ART-resistance, especially the haemoglobin trafficking pathway, which correlated with K13 mutations and their effect on the transcriptome [22, 23]. It was found that K13 mutations altered multiple intra-erythrocytic development programs, which impact the cell cycle, unfolded protein response, protein degradation, vesicular trafficking and mitochondrial metabolism [23]. The gene knockout of *Plasmodium*-specific putative phosphoinositide-binding protein (*Pf*PX1) was found to affect the haemoglobin trafficking pathway and made the parasite ART-resistant, suggesting that the haemoglobin trafficking pathway is an essential process for the ART-resistance mechanism. It was also shown that *Pf*PX1 binds to phosphatidylinositol-3-phosphate (PI3P), a molecule that has already been shown to be affected by K13 mutation [15, 24]. Taken together, different Fd and ARPS10 alleles might affect the isoprenoid synthesis pathway that leads to insufficient substrates for vesicle formation, especially the haemoglobin vesicular trafficking pathway which is also affected by the K13 mutation and consequently enhances parasite ART-resistance. A future experiment to assess the importance of the isoprenoid synthesis pathway would be to examine the ring survival rate of the K^MT^F^MT^A^MT^ parasite line in the presence of isopentenyl pyrophosphate (IPP), an isoprenoid precursor that can rescue parasites from lethal apicomplast dysfunction.

## Supplementary information

**Additional file 1: TableS1 **Oligonucleotides used to generate guide RNA, primers in PCR analysis ofparasite genetic modification and primers for sequence confirmation.

**Additional file 2: Table S2. **The sequences of designed elements (HR1-Recodonized part-HR2) of therepair plasmids for each mutagenesis.

**Additional file 3: Fig.S1. **Graphscomparing the percentage of the different stages and results of the statisticalanalysis using one-way ANOVA with Dunnett's multiple comparisons test of mock(DMSO), 20 nM DHA and 700 nM DHA treatments. T-S; trophozoite to schizontstage, R; ring stage. 

**Additional file 4: Fig. S2. **Sequence analysis demonstratingthe presence of fd-D193Y (upper) and arps10-V127M (lower) alleles in the MRA1240parasite line. The mutant codons are enclosed in thered box, compared with the 3D7 reference line.

## Data Availability

All plasmids and transgenic *P. falciparum* parasites described in this study are available upon request.
