# Supplementary material for: Apicoplast ribosomal protein S10-V127M enhances artemisinin resistance of a Kelch13 transgenic Plasmodium falciparum
Source: Malar J. 2022 Oct 27;21:302. doi: 10.1186/s12936-022-04330-3 (PMC9615251; doi:10.1186/s12936-022-04330-3)
Supplement: Supplementary file 1 — Additional file 1: TableS1 Oligonucleotides used to generate guide RNA, primers in PCR analysis ofparasite genetic modification and primers for sequence confirmation. [file 12936_2022_4330_MOESM1_ESM.docx]

**Supplementary Table S1** Oligonucleotides used to generate guide RNA, primers in PCR analysis of parasite genetic modification and primers for sequence confirmation.

| Oligo/ Primer name | Sequence 5’🡪 3' | Note |
| --- | --- | --- |
| k13-C580Y mutagenesis |  |  |
| k13guide_Oligo1 | ATTGACACACATAGCTGATGATCT | for Pfk13-g fragment construct |
| k13guide_Oligo2 | AAACAGATCATCAGCTATGTGTGT | for Pfk13-g fragment construct |
| K1 | GAAAGTGAAGCCTTGTTG | for genotype analysis of k13 and k13-C580Y sequence confirmation |
| K2 | CAAAAGCAACACACATAGC | for genotype analysis of k13-wild type |
| K3 | gATcTTgTTgTCgAAtGC | for genotype analysis of k13-C580Y |
| K4 | CTGCCACATTGTCAGATT | for k13C580Y sequence confirmation |
| fd-D193Y mutagenesis |  |  |
| fdguide_Oligo1 | ATTGTTGTGTGATTGAAACGCACA | for Pffd-g fragment construct |
| fdguide_Oligo2 | AAACTGTGCGTTTCAATCACACAA | for Pffd-g fragment construct |
| F1 | TAATATCTTTAAATATACTTCGCC | for genotype analysis of fd |
| F2 | TGTAGTTCGTCTTCCTTG | for genotype analysis of fd-wild type |
| F3 | CATaTaaTGTAacTCaTCcTC | for genotype analysis of fd-D193Y |
| F4 | GGTAGTTGTTCTACATGC | for fd-D193Y sequence confirmation |
| F5 | TCATTCCCCATTTCAATC | for fd-D193Y sequence confirmation |
| arps10-V127M mutagenesis |  |  |
| arps10guide_Oligo1 | ATTGCTTTTTGCGATCTCCCCATG | for Pfarps10-g fragment construct |
| arps10guide_Oligo2 | AAACCATGGGGAGATCGCAAAAAG | for Pfarps10-g fragment construct |
| arps10HR1_F (a4) | gacattttgttttgttagTTGG | for Pfarps10HR1 amplification and arps10-V127M sequence analysis |
| arps10HR1_R | gtttcattttagATTTACCTTcTTaaGgagTCCaCAcaTG | for Pfarps10HR1 amplification |
| arps10HR2_F | TcTTaaGgagTCCaCAcaTGGATAAAGACAGTAGAGAGC | for Pfarps10HR2 amplification |
| arps10HR2_R | CCAACTTATATAACGCTCAAG | for Pfarps10HR2 amplification |
| a1 | TTTTGCGATCTCCCCATG | for genotype analysis of arps10-wild type |
| a2 | CTAAGGGGAATTTGTATAATTCG | for genotype analysis of arps10 |
| a3 | TaaGgagTCCaCAcaTGG | for genotype analysis of arps10-V127M |
