# Supplementary material for: Apicoplast ribosomal protein S10-V127M enhances artemisinin resistance of a Kelch13 transgenic Plasmodium falciparum
Source: Malar J. 2022 Oct 27;21:302. doi: 10.1186/s12936-022-04330-3 (PMC9615251; doi:10.1186/s12936-022-04330-3)
Supplement: Supplementary file 2 — Additional file 2: Table S2. The sequences of designed elements (HR1-Recodonized part-HR2) of therepair plasmids for each mutagenesis. [file 12936_2022_4330_MOESM2_ESM.docx]

**Supplementary Table S2** The sequences of designed elements (HR1-Recodonized part-HR2) of the repair plasmids for each mutagenesis.

| Design repair element sequences for k13 C508Y mutagenesis | GCGTATGTGTACACCTATGTCTACCAAAAAAGCTTATTTTGGAAGTGCTGTATTGAATAATTTCTTATACGTTTTTGGTGGTAATAACTATGATTATAAGGCTTTATTTGAAACTGAGGTGTATGATCGTTTAAGAGATGTATGGTATGTTTCAAGTAATTTAAATATACCTAGAAGAAATAATTGTGGTGTTACGTCAAATGGTAGAATTTATTGTATTGGGGGATATGATGGCTCTTCTATTATACCGAATGTAGAAGCATATGATCATCGTATGAAAGCATGGGTAGAGGTGGCACCTTTGAATACCCCaAGgTCtTCtGCaATGTaTGTaGCaTTcGAcAAcAAgATcTATGTCATTGGTGGAACTAATGGTGAGAGATTAAATTCTATTGAAGTATATGAAGAAAAAATGAATAAATGGGAACAATTTCCATATGCCTTATTAGAAGCTAGAAGTTCAGGAGCAGCTTTTAATTACCTTAATCAAATATATGTTGTTGGAGGTATTGATAATGAACATAACATATTAGATTCCGTTGAACAATATCAACCATTTAATAAAAGATGGCAATTTCTAAATGGTGTACCAGAGAAAAAAATGAATTTTGGAGCTGCCACATTGTCAGATTCTTATATAATTACAGGAGGAGAAAATGGCGAAGTTCTAAATTCATGTCATTTCTTTTCACCAGATACAAATGAATGGCAG |
| --- | --- |
| Design repair element sequences for fd D193Y mutagenesis | TATTTTTGAATTTCCTAAGTAATAATCAGCTAGCTAATTCTAATAAACAAACATGTTTTTTTAAGAGCAATATAAAAAGTAGTATATCAAATATCGATAATTATGATTATATAAGAAAACGTTATATCAATACATCTAATAAAAATAAATTATTTTATAATATAACATTAAGAACAAATGATGGAGAAAAAAAAATCGAATGTAATGAAGATGAATATATATTAGATGCTAGTGAAAGACAGAATGTTGAATTACCATATAGTTGTAGGGGAGGTAGTTGTTCTACATGCGCAGCAAAATTAGTCGAAGGAGAAGTAGATAATGATGATCAAAGTTATTTAGATGAAGAGCAAATAAAGAAAAAATATATTCTTTTGTGTACATGTTATCCTAAATCGGATTGcGTaATaGAgACcCAtAAaGAgGAtGAgtTACAttAtATGTAAtttgtcctcaatcaaaaatatgtgtaattatatatatatatatatgtaaatatatgtatatttatttattatttcattttattttattttttttggtatggatatgattgaaatggggaatgataaaatttcctaatatttttctcaattaaaaatttttttttttattttttattttatataaatatttaaacagataaaaaaaaaaaaaaaaaaaaaaaaaaaaacatataattctttaaatattaatatatgtctctagacattttatacataaaaaaaagaaaagttaaaaaaaaaacaaatataatgtattttaattatacacatatatatatgtgtatgtattaacttcgctaccgtttttatatataattattatataattttaaaaactact |
| Design repair element sequences for arps10 V127M mutagenesis | gacattttgttttgttagTTGGATAAAGTAAATAATGATTTAAAAAAGAAAGATATAATAAAAAAATGGACTGAGAATTATCATTTGCGAATAATATTAAGTTCTTATTTTTCTGATCATTTACAAAAAGCTGTTTTTAATGTTAAGGAAAAATTAAGTCAATATCCTCAGTTTATTGTAGCAGGCCCAATTCCCCAAAAGACAATAAGAAAGAGgtaacatatatatttgtgaagataaaaacaaataaataaataaatatatatatatatatatatatatatatatatatatatatatatatatgtacacaatattatgtttcattttagATTTACCTTcTTaaGgagTCCaCAcaTGGATAAAGACAGTAGAGAGCAATTCGAAATAAAACAGTACTCTTGCAAATTAGATATTTTTTTGAACTCATCTGTTCCTATAAAAAATTCTGAATgtaagcagaataaattatacacaaaagaatatatattcatttttatttattcactttatatatgtttgtatgtatgtatgtatgtttttcaccttccagttgtaaatttcttatcagTGAAATTACCCAGATTTGTGGGTTTTGAATACTACTTTGAAGAGAATTATAAAGGTTTGTCAAAAGAAGAAGTACAAAAATTAAAGAAGAAAAAATATGTCAGTAAATATTATACCAACTTATATAACGCTCAAG |

The highlighted sequences are the recodonized regions, with bases in lower case those that are substituted and the mutated codon underlined.
