# Supplementary material for: Apicoplast ribosomal protein S10-V127M enhances artemisinin resistance of a Kelch13 transgenic Plasmodium falciparum
Source: Malar J. 2022 Oct 27;21:302. doi: 10.1186/s12936-022-04330-3 (PMC9615251; doi:10.1186/s12936-022-04330-3)

**Supplementary Figure S1** Graphs comparing the percentage of the different stages and results of the statistical analysis using one-way ANOVA with Dunnett's multiple comparisons test of mock (DMSO), 20 nM DHA and 700 nM DHA treatments. T-S; trophozoite to schizont stage, R; ring stage.


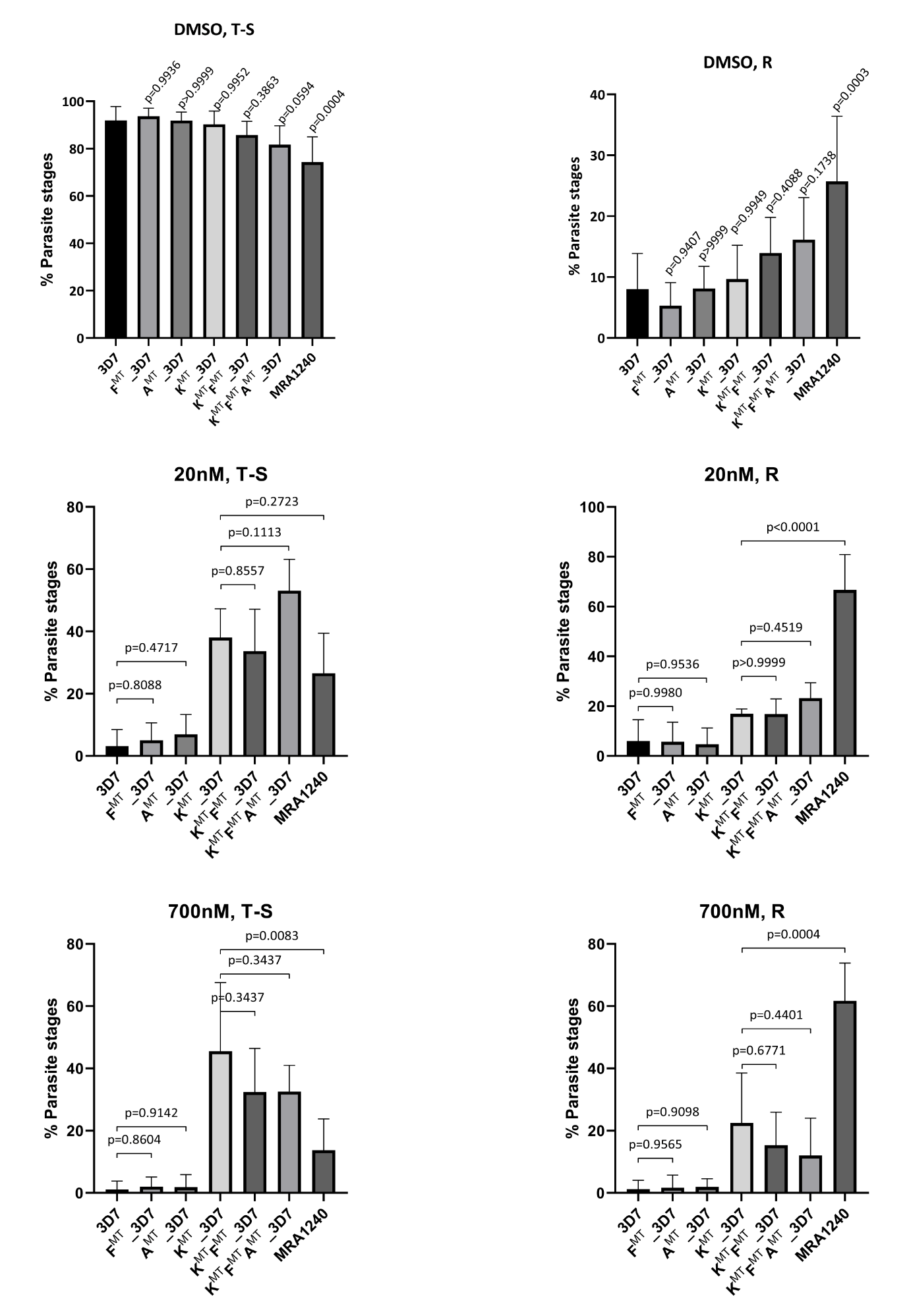

Supplement: Supplementary file 3 — Additional file 3: Fig.S1. Graphscomparing the percentage of the different stages and results of the statisticalanalysis using one-way ANOVA with Dunnett's multiple comparisons test of mock(DMSO), 20 nM DHA and 700 nM DHA treatments. T-S; trophozoite to schizontstage, R; ring stage. [file 12936_2022_4330_MOESM3_ESM.docx]
