# Supplementary material for: Apicoplast ribosomal protein S10-V127M enhances artemisinin resistance of a Kelch13 transgenic Plasmodium falciparum
Source: Malar J. 2022 Oct 27;21:302. doi: 10.1186/s12936-022-04330-3 (PMC9615251; doi:10.1186/s12936-022-04330-3)
Supplement: Supplementary file 4 — Additional file 4: Fig. S2. Sequence analysis demonstratingthe presence of fd-D193Y (upper) and arps10-V127M (lower) alleles in the MRA1240parasite line. The mutant codons are enclosed in thered box, compared with the 3D7 reference line. [file 12936_2022_4330_MOESM4_ESM.docx]

**Supplementary Figure S2** Sequence analysis demonstrating the presence of fd-D193Y (upper) and arps10-V127M (lower) alleles in the MRA1240 parasite line. The mutant codons are enclosed in the red box, compared with the 3D7 reference line.

**
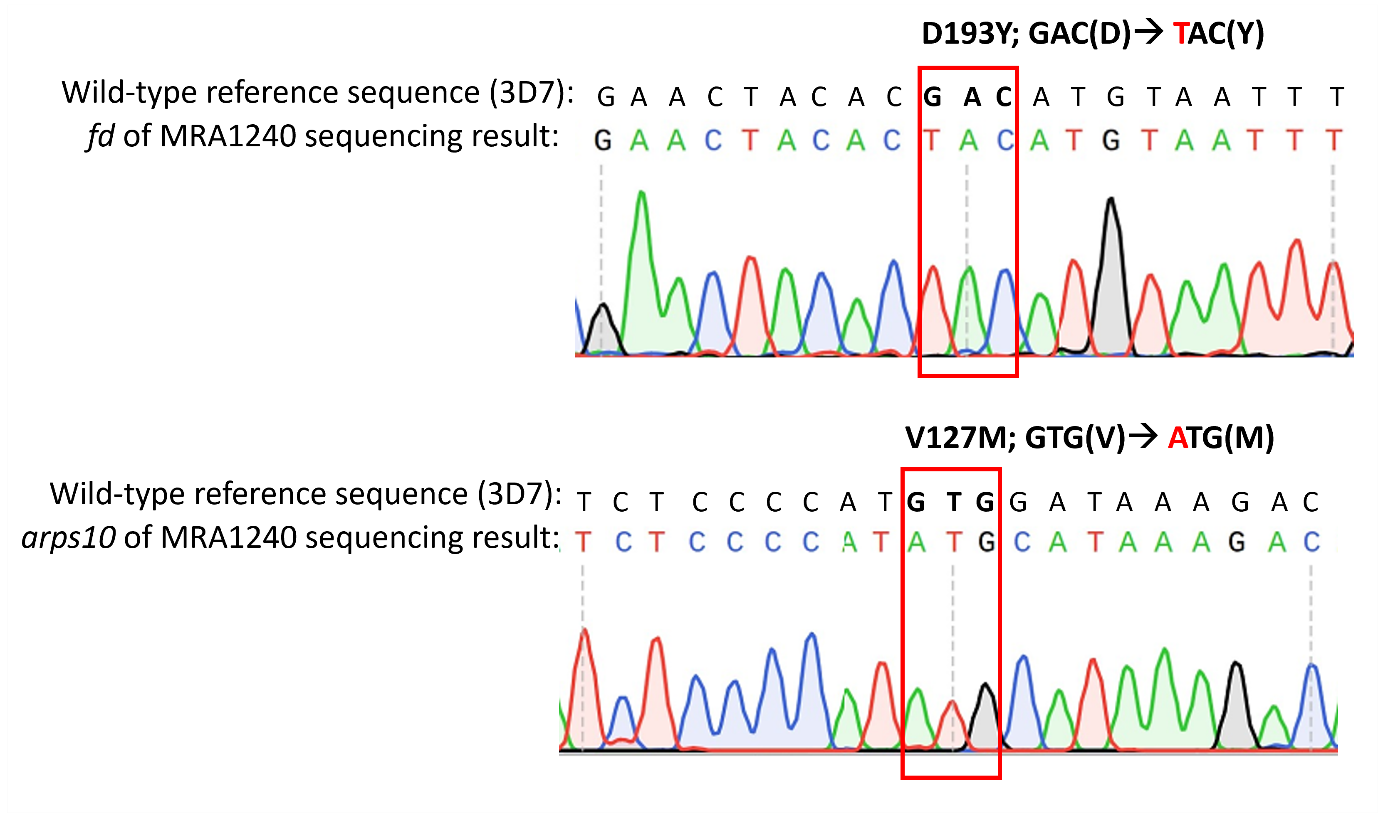
**
